# Transcriptome profiling reveals the genes involved in tuberous root expansion in Pueraria (*Pueraria montana* var. *thomsonii*)

**DOI:** 10.1186/s12870-023-04303-x

**Published:** 2023-06-26

**Authors:** Xiao Xufeng, Hu Yuanfeng, Zhang Ming, Si Shucheng, Zhou Haonan, Zhu Weifeng, Ge Fei, Wu Caijun, Fan Shuying

**Affiliations:** 1 College of Agronomy, Jiangxi Agricultural University, Nanchang, 330045 China; 2 Department of Biological Engineering, Jiangxi Biotech Vocational College, Nanchang, 330200 China; 3 College of Pharmacy, Jiangxi University of Chinese Medicine, Nanchang, 330004 China

**Keywords:** Pueraria, Tuberous root expansion, Transcriptome, Molecular mechanism

## Abstract

**Background:**

Pueraria is a dry root commonly used in Traditional Chinese Medicine or as food and fodder, and tuberous root expansion is an important agronomic characteristic that influences its yield. However, no specific genes regulating tuberous root expansion in Pueraria have been identified. Therefore, we aimed to explore the expansion mechanism of Pueraria at six developmental stages (P1–P6), by profiling the tuberous roots of an annual local variety “Gange No.1” harvested at 105, 135, 165, 195, 225, and 255 days after transplanting.

**Results:**

Observations of the tuberous root phenotype and cell microstructural morphology revealed that the P3 stage was a critical boundary point in the expansion process, which was preceded by a thickening diameter and yield gain rapidly of the tuberous roots, and followed by longitudinal elongation at both ends. A total of 17,441 differentially expressed genes (DEGs) were identified by comparing the P1 stage (unexpanded) against the P2–P6 stages (expanded) using transcriptome sequencing; 386 differential genes were shared across the six developmental stages. KEGG pathway enrichment analysis showed that the DEGs shared by P1 and P2–P6 stages were mainly involved in pathways related to the “cell wall and cell cycle”, “plant hormone signal transduction”, “sucrose and starch metabolism”, and “transcription factor (TF)”. The finding is consistent with the physiological data collected on changes in sugar, starch, and hormone contents. In addition, TFs including bHLHs, AP2s, ERFs, MYBs, WRKYs, and bZIPs were involved in cell differentiation, division, and expansion, which may relate to tuberous root expansion. The combination of KEGG and trend analyses revealed six essential candidate genes involved in tuberous root expansion; of them, CDC48, ARF, and EXP genes were significantly upregulated during tuberous root expansion while INV, EXT, and XTH genes were significantly downregulated.

**Conclusion:**

Our findings provide new insights into the complex mechanisms of tuberous root expansion in Pueraria and candidate target genes, which can aid in increasing Pueraria yield.

**Supplementary Information:**

The online version contains supplementary material available at 10.1186/s12870-023-04303-x.

## Introduction

Pueraria is the dry root of leguminous *Pueraria montana* var. *lobata* (Willdenow.) Maesen & S. M. Almeida ex Sanjappa et Predeep or *Pueraria montana* var. *thomsonii* (Bentham) M. R. Almeida; the former is well known as Gegen (Chinese name) and the latter known as Fengen (Chinese name) in Traditional Chinese Medicine (TCM) [1]. Pueraria, one of the earliest medicinal herbs used in China [2], is native to Southeast Asia and has been employed as a traditional Chinese medicine, food source, and fodder for thousands of years. Pueraria has been used to treat fever, diarrhea, emesis, cardiac dysfunction, liver injury, weight loss, and toxicosis in humans [3]. In the Pharmacopoeia of the People’s Republic of China (PPRC), there are two primary varieties of *Pueraria*: *Pueraria montana* var. *lobata* (Willdenow.) (*Pueraria montana* var. *lobatae* Radix, PLR), and *Pueraria montana* var. *thomsonii* (*Pueraria montana* var. *thomsonii* Radix, PTR). Historically, the herbal industry and many traditional Chinese medicine dispensers/practitioners believed that these species had similar effects. Therefore, they have been treating these herbs as functionally the same, making no distinction in species used for manufacturing and clinical practice. More recently, PTR has been increasingly used in cooking, emerging as a specialty vegetable that is gradually becoming recognized by the market [4]. Thus, we suggest that the food value of PTR may be greater than its medicinal use. For use as an authentic herb, Pueraria has been thoroughly studied for its chemical composition, pharmacological mechanisms, and clinical applications; however, little research has been conducted to explore its food value, especially at the molecular level.

Since the late 1950s, molecular pharmacognosy of Pueraria, including molecular identification, transcriptome sequencing, cloning, and synthesis of functional genes, has been gradually reported. Research has mainly focused on isoflavone biosynthesis [5–7], such as puerarin. Recently, using PacBio-RA-II and Ilumina platforms for simultaneous sequencing analysis of Pueraria [8], 3’- methyltransferase, isoflavone-specific 4’- O methyltransferase, and other genes were more highly expressed in PTR than in PLR. Further, using high-throughput sequencing technology, Nithiwat et al. identified 21 genes that may be involved in isoflavone biosynthesis from the combined tissues of young leaves, mature leaves, tuber cortex, and peel tuber of Thailand Pueraria (*Pueraria candollei* var. *Mirifica*) [9]. Additionally, Wang et al. performed molecular cloning and functional characterization of PIUGT43 (a novel glucosyltransferase from PLR). Biochemical analysis showed that PIUGT43 promotes C-glycosylation, which converts soybean sapogenins to puerarin, and that PIUGT43 is active against isoflavonoid soybean sapogenins and genistein. Therefore, the lack of activity against other potential receptors, including flavonoids, confirmed the role of PIUGT43 in puerarin biosynthesis [10].

In addition to isoflavones, the physicochemical properties of Pueraria starch, such as viscosity, thermal instability, retrogradation, gelatinization, and digestibility, comprise current topics of interest in research of Pueraria’s food value [11, 12]. Tuberous root development in Pueraria is a complex process regulated by multiple genes, whose expression is determined by light, temperature, hormones, carbon metabolites, cell expansion and division, and TFs [13, 14]. The ADP-glucose pyrophosphorylase (*AGPase*) gene, for example, a key enzyme in the starch synthesis of Pueraria, affects the composition of starch by regulating changes in its activity, root length, root diameter, weight per plant, and yield [15]. Recent research has focused only on the molecular mechanism of tuberous root expansion during Pueraria growth and development. For example, Liu et al. screened five specific sequences for controlling tuberous root enlargement in PTR by cDNA-AFLP, which are generally involved in signal transduction, stress resistance response, electron transport, and photosynthetic metabolism [16]. To date, few genes related to tuberous root formation have been identified, and no specific genes regulating tuberous root expansion in Pueraria have been identified. Therefore, more research is needed to reveal the molecular mechanism of Pueraria tuberous root expansion.

In production, tuberous root expansion is a determinant of the yield of PTR cultivated as a medicinal and edible vegetable. However, tuberous root expansion is a complex biological process, and its mechanism is still unclear. It was found that some specific genes and proteins associated with starch and phytohormone synthesis, as well as various TFs, are involved in tuberous root formation and expansion [17–19], but there are many genes should be found at the transcriptional level. In this study, we thoroughly investigated the transcriptomic mechanism of tuberous root expansion in PTR. Since we did not find any reports of Pueraria tuberous root expansion, we first observed tuberous root changes during the developmental process of “Gange No. 1”, an annual local PTR variety in Jiangxi province. After then, we referred to the sampling time of other metamorphic organ expansion crops, such as sweet potato [20], potato [21], and yam [22], and divided the tuberous root expansion process of PTR into six stages. Next, we compared the difference in morphological indices, sugar, starch and flavonoid accumulation, endogenous hormone content, and transcriptomes of six different developmental phases. We aimed to explore the gene regulatory networks associated with Pueraria tuberous root expansion and to mine the key genes that mediate tuberous root expansion. The findings could directly guide PTR production, improve the technical level of PTR cultivation, and help to address practical problems in the production process of PTR, such as increasing production or precocious cultivation. Additionally, this study laid the foundation for further research on the puffed mechanism at the molecular gene level, potentially guiding the breeding of PTR to maximize its food and medicinal values.

## Results

### The phenotype of tuberous root expansion

**Fig. 1:**
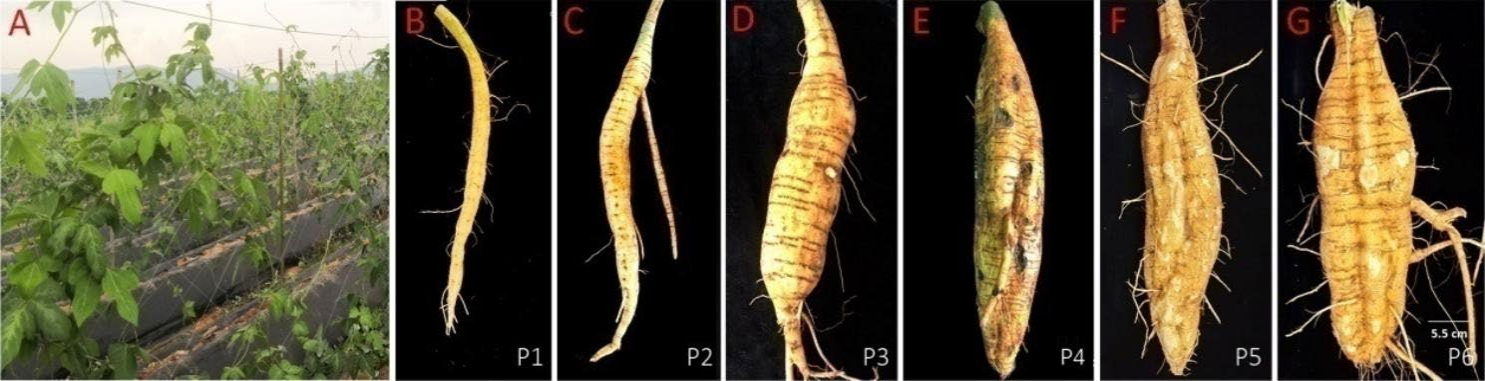
The phenotype of different developmental stages of PTR tuberous root. (**A**) Field cultivation of “Gange No. 1” in May; (**B**) P1 represents the unexpanded stage at 105 d after transplanting; (**C**) P2 represents the initial-expanded stage at 135 d after transplanting; (**D**) P3 represents the mid-expanded stage at 165 d after transplanting; (**E**) P4 represents the mid-late expanded stage at 195 d after transplanting; (**F**) P5 represents the late-expanded stage at 225 d after transplanting; (**G**) P6 represents the end-expanded stage at 255 d after transplanting

Samples were collected from field-grown cultivar “Gange No. 1” during its unexpanded and expanded stages (Fig. 1A). From Fig. 1, we did not find any expansion changes in the tuberous root at the P1 stage and defined it as an unexpanded stage (Fig. 1B). Tuberous root expansion initiated from the P2 stage, and went through five expansion periods in total: initial-expansion (the P2 stage) (Fig. 1C), mid-expansion (the P3 stage) (Fig. 1D), mid-late expansion (the P4 stage) (Fig. 1E), late-expansion (the P5 stage) (Fig. 1F), and end-expansion (the P6 stage) (Fig. 1G). We found significant phenotypic differences between the P1 stage (unexpanded stage) and the P2–P6 stages (expanded stages) in multiple tuberous root traits. First, the transverse diameter of the tuberous roots rapidly increased from P1 to P3. Until the P3 stage, the transverse diameter increased by 7.39 cm compared to that of the P1 stage (*p* < 0.05). Meanwhile, tuberous root weight also increased rapidly from P1 to P3, and compared to that of the P1 stage, root weight increased by 708.2 g in P3 (*p* < 0.05) (Table 1).

**Table 1:** Statistical analysis of root-related traits in different development stages of PTR

Different growth phases	Transverse diameter (cm)	Longitudinal diameter (cm)	Fresh weight (g)
P1	1.28 ± 0.26c	21.83 ± 3.33c	99.80 ± 48.88d
P2	3.50 ± 0.50b	22.83 ± 1.89c	306.52 ± 47.28c
P3	8.67 ± 0.76a	27.00 ± 8.00bc	808.00 ± 90.24b
P4	8.74 ± 1.02a	33.03 ± 5.53b	876.62 ± 44.56b
P5	8.85 ± 0.56a	34.50 ± 2.18b	949.00 ± 163.72ab
P6	9.02 ± 0.73a	50.45 ± 1.24a	1094.48 ± 15.98a

The longitudinal diameter of tuberous roots changed rapidly after the P3 stage, increasing by 17.42 cm (*p* < 0.05) from P4 to P6. Tuberous root weight gain slowed from the P4 stage onwards, with root weight only increasing by 217.86 g at P6 (*p* < 0.05) (Table 1). These results indicate that the P3 stage might be an essential boundary point at which tuberous roots of PTR grow faster firstly, especially in terms of lateral growth thickening and weight gain. In contrast, the growth was relatively slow in the later stages, mainly in the longitudinal elongation at both ends.

### Microstructural changes in tuberous root expansion

**Fig. 2:**
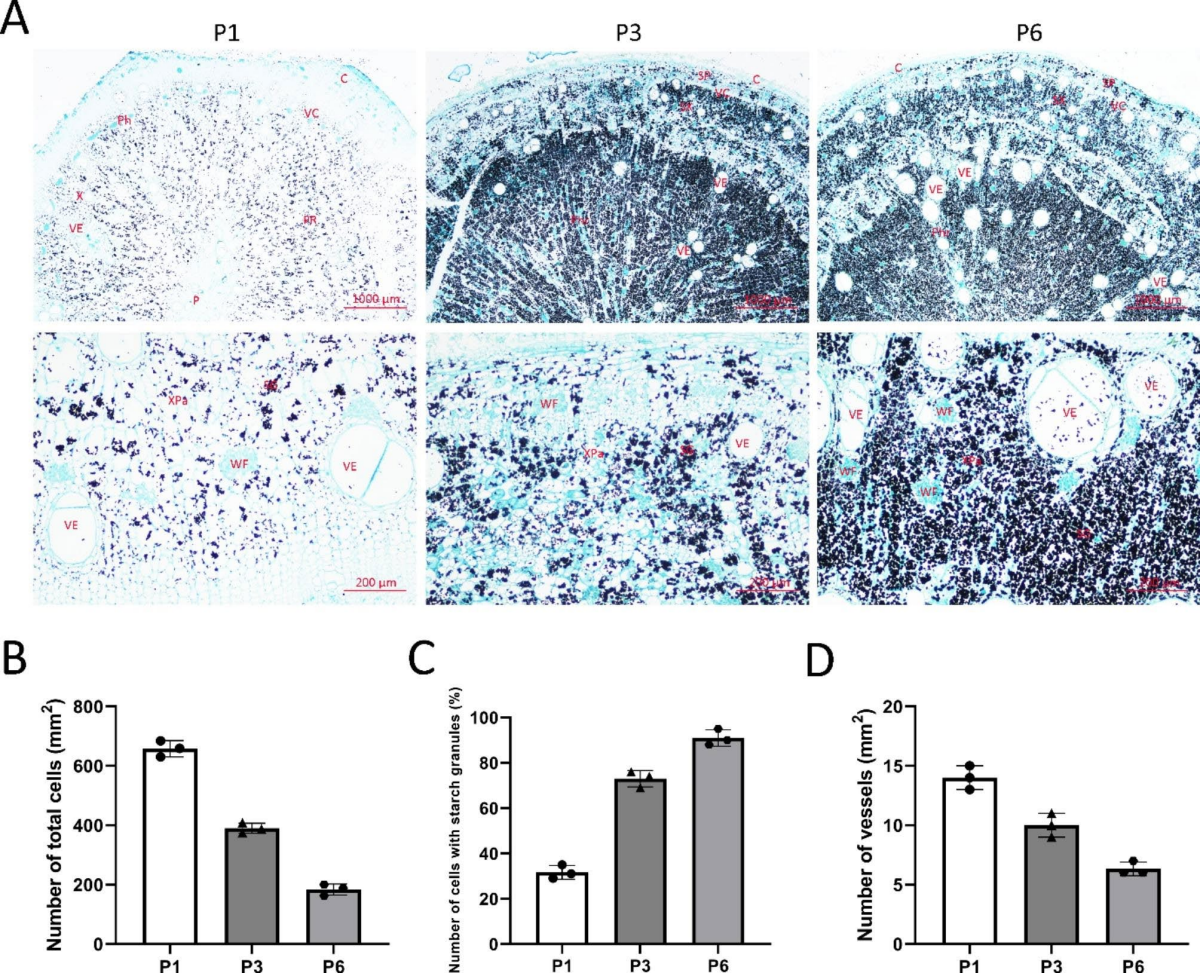
Cytological observation in different development stages. Tuberous roots from P1, P3, and P6. C: cortex; VC: vascular cambium; P: pith; PR: pith ray; Ph: phloem; Phr: phloem ray; X: xylem; SP: secondary phloem; SX: secondary xylem; XPa: xylem parenchyma; SG: starch granules; WF: wood fiber. (**A**) Cytological observation of P1, P3, and P6, respectively; (**B**) Number of total cells per mm^2^; (**C**) Number of cells with starch granules per mm^2^; (**D**) Number of vessels per mm^2^. Bar, 1000 μm and 200 μm

Based on the above experimental results, we considered that the changes in tuberous roots at the P1, P3, and P6 stages were representative, and therefore carried out cytological observations on the tuberous roots at these three developmental stages. The phloem (Ph), vascular cambium (VC), xylem (X), vessel (VE), and pith (P) could be distinguished among the three developmental stages, with the most noticeable distinction between xylem and phloem tissues (Fig. 2).

In the P1 stage, the shape of the vessels was regular and well-developed, and the lumen diameter was significantly larger than that of thin-walled cells in the xylem (Fig. 2A). The average number of cells per unit area (mm^2^) reached 656 (Fig. 2B), and the number of intracellular amyloplasts was small and sparsely distributed, mostly at the edges of cells (Fig. 2A and C). In the P3 stage, the proportion of the secondary xylem increased along with tuberous root expansion (Fig. 2A). The number of cells per mm^2^ decreased and the number of vessels was more differentiated than that in the P1 stage (Fig. 2B and D). The thin-walled cells rapidly became larger, the shape from the original sub-circular constantly increasing mutual extrusion into irregular polygonal and the amyloplasts increased within the cells (Fig. 2C). Additionally, from the middle column of the pith, secondary xylem, newly formed xylem, and starch grains showed a distribution pattern of radial-neatly, dense-disorganized sparse. At P6, the number of cells per mm^2^ still decreased and the vessels were regular and well developed (Fig. 2B and D), with a wide distribution. At this time, starch mainly accumulated in the parenchyma cells of the secondary xylem, though a few starch granules were also distributed in secondary phloem cells (Fig. 2A). Based on histological observations, tuberous root expansion before the P3 stage may be mainly related to the increase in the number and size of xylem thin-walled cells. In contrast, tuberous root expansion after P3 may be mainly related to the stretch in cell volume. Therefore, starch granules accumulate through PTR tuberous root expansion.

### Sugar, starch and flavonoid accumulation

**Fig. 3:**
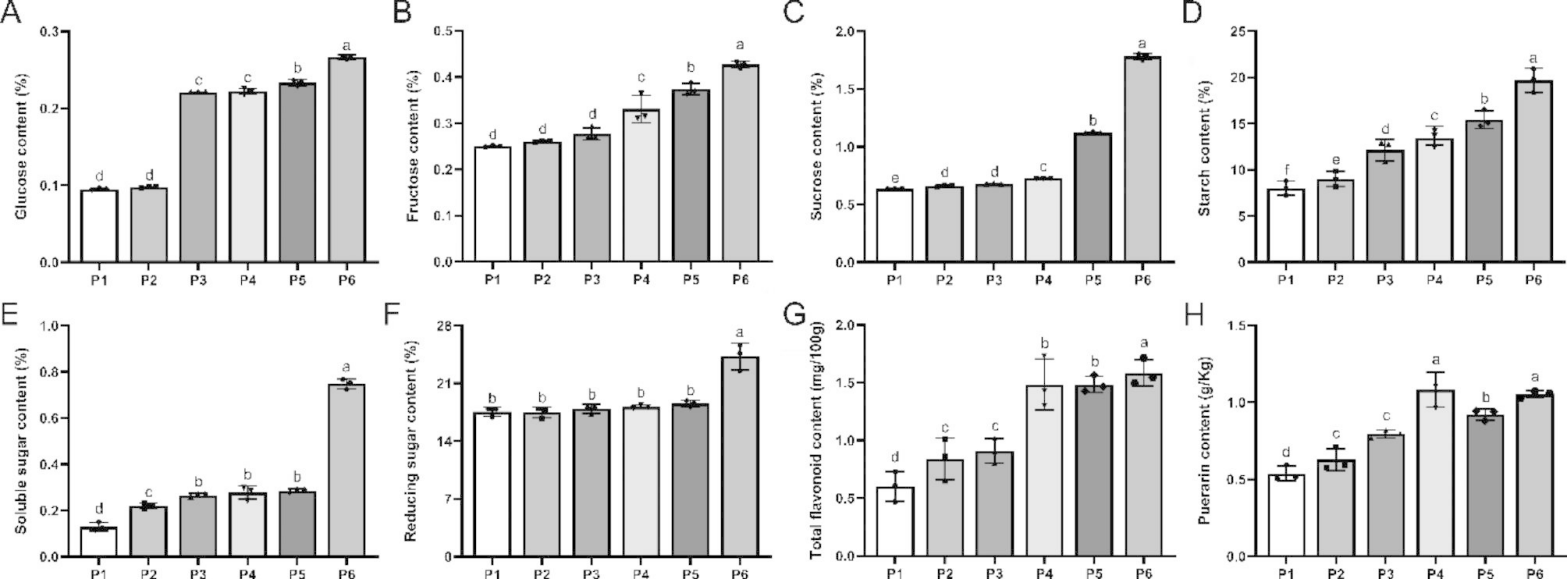
Dynamic changes of sugar, starch, and flavonoid contents in different developmental stages. (**A**) Glucose content; (**B**) Fructose content; (**C**) Sucrose content; (**D**) Starch content; (**E**) Soluble sugar content; (**F**) Reducing sugar content; (**G**) Total flavonoid content; (**H**) Puerarin content

With the tuberous root expansion of Pueraria, the dynamic changes in glucose, fructose, sucrose, and starch content showed different degrees of increase (Fig. 3A–D). Among them, fructose and starch content showed steady growth throughout development, while glucose and sucrose contents fluctuated rapidly in the period from P2–P3 and P4–P6, respectively. Soluble sugars increased from P5 to P6, though there was a slight increase in other stages (Fig. 3E); while reducing sugar content showed a slow increasing trend, with a significant increase at the period from P5–P6 (*p* < 0.05) (Fig. 3F). In addition, total flavonoid content showed a significant increase with the expansion of the PTR tuberous root (Fig. 3G); while puerarin content increased in the P1–P4 stages, but showed fluctuations occur in the P5 stages (*p* < 0.05) (Fig. 3H).

### Endogenous hormone content change

**Fig. 4:**
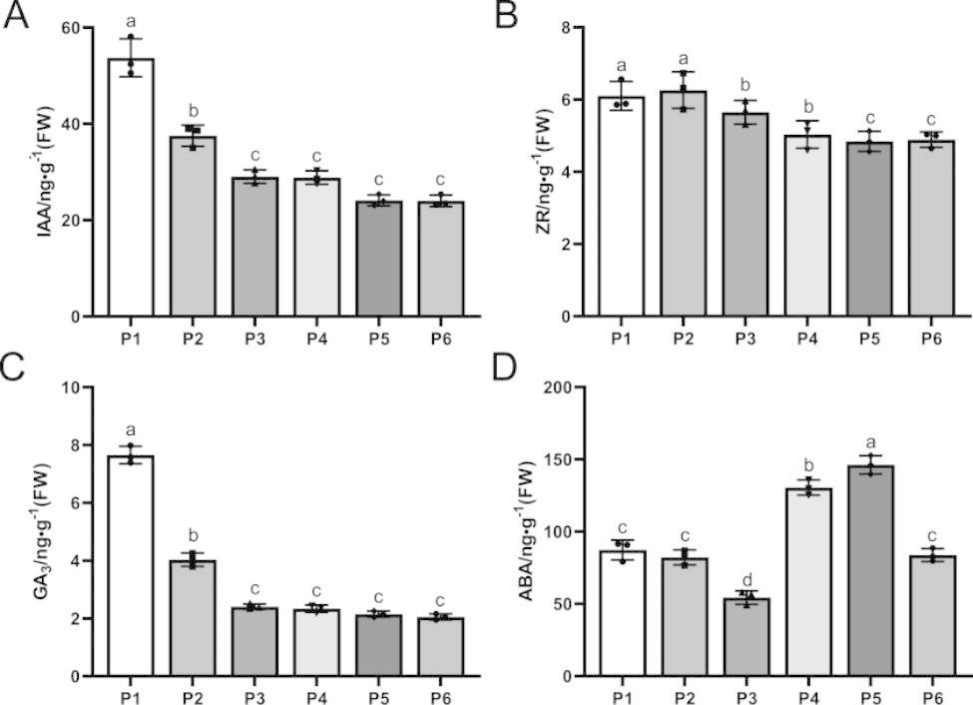
Dynamic changes of endogenous hormone contents in different developmental stages. (**A**) IAA content; (**B**) ZR content; (**C**) GA_3_ content; (**D**) ABA content

IAA, ZR, and GA3 all decreased during tuberous root expansion, with ZR decreasing more gradually than the others. In contrast, IAA and GA3 decreased more during the P1–P3 stages and tended to level off (Fig. 4A–C). ABA content showed a “decrease–increase–decrease” trend in the process of tuberous root expansion, characterized by a significant decrease before the P3 stage, followed by a rapid increase during the P4 and P5 stages, reaching a maximum in the P5 stage, and a final decline in the P6 stage (Fig. 4D).

### Transcriptome data acquisition

**Fig. 5:**
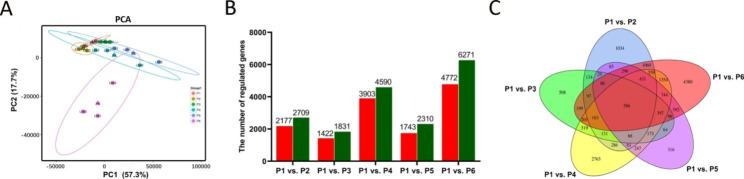
Comparative transcriptome analysis of P1 stage with P2, P3, P4, P5, and P6 stages of PTR. (**A**) Scores scatter plot of root transcriptome in unswollen root and swollen root as determined by PCA. (**B**) The number of regulated genes between the P1 and P2–P6 stage; the red color represents the upregulated genes and the green color represents the downregulated genes. (**C**) Venn diagram of regulated genes among different developmental stages

We generated 18 cDNA libraries from unexpanded tuberous roots (P1) and expanded tuberous roots at different stages (P2, P3, P4, P5, and P6) in PTR. Based on the Illumina sequencing results, we assessed gene expression levels for each replicate using principal component analysis (PCA) (Fig. 5A). In this study, two main components, PC1 and PC2, were extracted at 57.3% and 17.7%, respectively, with a cumulative contribution rate of 75.0%. In the PCA score plot, the repeated samples were compactly gathered, indicating that the samples were reproducible, and the data were ready for subsequent analysis. In addition, the P4, P5, and P6 samples clustered far from the P1 samples, indicating that tuberous root expansion induced changes in gene expression between stage types. All Unigenes under the five different groups were annotated and compared using the COG, GO, KEGG, KOG, Swiss-Prot, and eggnog databases (Table 2). We identified 17,441 differentially expressed genes (DEGs) in the six comparison groups. Genes with a false discovery rate (FDR) < 0.05 and an absolute value of log2 ratio ≥ 1 were selected as DEGs. An additional movie file (Table S1) shows this observation in greater detail. The results of the DEGs expression analysis showed that compared with that of the unexpanded stage, the number of differential genes in an expanded stage was relatively high at 255 days (P6 stage) of tuberous root expansion, and the proportion of downregulated genes was higher than that of upregulated genes in all comparison groups (Fig. 5B). Finally, only 386 DEGs (Table S2) were common to all six comparison groups (Fig. 5C).

**Table 2:** Reference tag database and major characteristics of DEG libraries

Group	Annotated	COG	GO	KEGG	KOG	Swiss-Prot	eggNOG
P1 vs. P2	4780	2268	4002	1898	2636	3761	4463
P1 vs. P3	3177	1429	2623	1275	1743	2458	3167
P1 vs. P4	8239	3571	6809	3435	4904	6220	8219
P1 vs. P5	3931	1738	3261	1518	2128	3059	3912
P1 vs. P6	10,723	4753	8876	4385	6177	8121	10,695

### GO and KEGG enrichment analyses of DEGs

To further determine the main biological functions of all DEGs shared by the unexpanded and expanded stages, functional annotation was performed by mapping all the common DEGs to GO terms in the GO database. GO enrichment analysis was implemented using a Bonferroni-corrected *p* ≤ 0.05. Among the annotated 33,348 Unigenes selected to predict functions by GO annotation, we identified 4,002, 2,623, 6,809, 3,261, and 8,876 DEGs in P1 vs. P2, P1 vs. P3, P1 vs. P4, P1 vs. P5, and P1 vs. P6, assigned to at least one GO term classified into 55, 50, 64, 53, and 69 groups, including 23, 20, 25, 21, and 27 biological processes, 16, 14, 18, 16, and 18 molecular functions, and 16, 16, 21, 16, and 24 cellular components, respectively (Table S3).

Among the DEGs between P1 vs. P2, the “oxidation-reduction process” and “plant-type cell wall organization” were the major terms for biological process, the “cytosolic large ribosomal subunit” and “extracellular region” were the major terms for cellular components, and the “heme binding” was the most represented molecular function term. Between P1 vs. P3 group, the “oxidation-reduction process” was the major term for biological process, the “cytosolic large ribosomal subunit” and “cytosolic small ribosomal subunit” were the major terms for cellular component, and the “structural constituent of the ribosome” was the most represented molecular function term. Between P1 vs. P4 group, the “oxidation-reduction process” and “flavonoid biosynthetic process” were the major terms for biological process, “extracellular region” and “cytosolic large ribosomal subunit” were the major terms for cellular component, and “structural constituent of the ribosome” and “heme binding” were the most represented molecular function terms. Between P1 vs. P5 group, the “oxidation-reduction process” was the major term for biological process, “cytosolic large ribosomal subunit” was the major term for cellular component, and “structural constituent of the ribosome” and “heme binding” were the most represented molecular function terms. Between P1 vs. P6 group, the “plant-type cell wall organization” and “oxidation-reduction process” were the major terms for biological process, “cell wall” was the major term for cellular components, and “structural constituent of the ribosome” was the most represented molecular function term. Notably, the enrichment of DEGs in the P3 stage showed a significant increase in the starch biosynthetic and glycogen metabolic/biosynthetic processes. In contrast, at the P4 stage, the enrichment of DEGs was significantly increased in the flavonoid biosynthetic process.

**Fig. 6:**
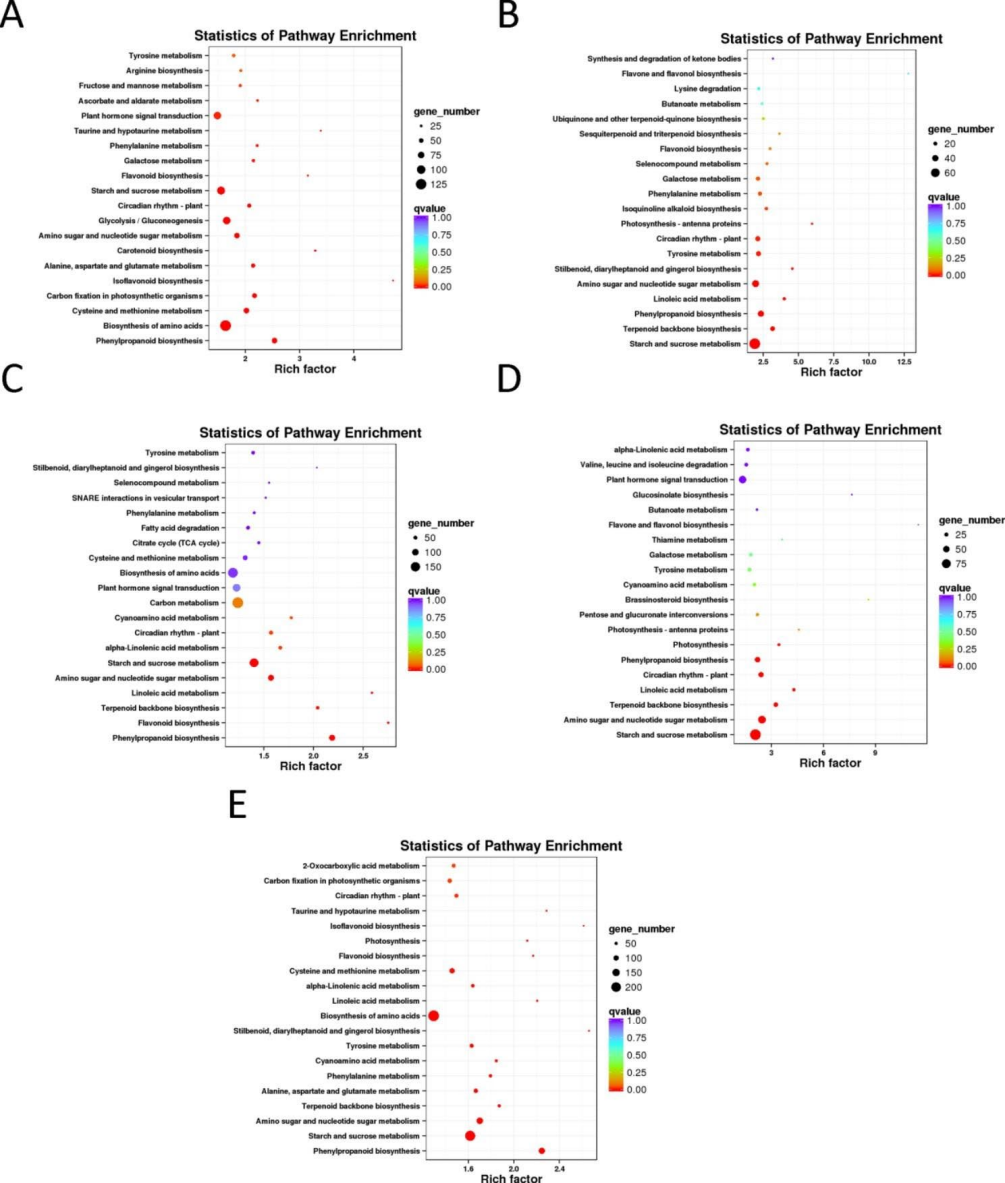
The top 20 KEGG enrichment pathways between the P1 stage and other expansion stages were obtained by the KEGG database (http://www.kegg.jp/kegg/kegg1.html) analysis. (**A–E**) P1 vs. P2, P1 vs. P3, P1 vs. P4, P1 vs. P5, and P1 vs. P6. They-axis is the pathway, and the x-axis is the percentage of this pathway of the total Rich Factor. The color depth represents the q-value. The darker the color, the smaller the q-value and the higher the enrichment degree. The size of the dots indicated the number of DEGs in this pathway

We performed a pathway enrichment analysis using the KEGG database to further determine the metabolic or signal transduction pathways in which common DEGs may participate in tuberous root expansion. A total of 1,130 (P1 vs. P2), 771 (P1 vs. P3), 1,904 (P1 vs. P4), 860 (P1 vs. P5), and 2,513 (P1 vs. P6) DEGs were assigned to 115, 116, 126, 118, and 125 pathways, respectively, by KEGG pathway enrichment (q ≤ 0.05).Additionally, 11, 10, 11, and 14 pathways were identified as significantly enriched in P1 vs. P2, P1 vs. P3, P1 vs. P4, P1 vs. P5, and P1 vs. P6, respectively (Table 3). Among them, the “biosynthesis of amino acids (ko01230)”, “carbon metabolism (ko01200)”, and “starch and sucrose metabolism(ko00500)” were the top three represented path ways among the DEGs between P1 and P2 (Fig. 6A). “Starch and sucrose metabolism (ko00500)”, “carbon metabolism (ko01200)”, and “plant hormone signal transduction (ko04075)” were the top three path ways among DEGs between P1 and P3 (Fig. 6B). The “carbon metabolism (ko01200)”, “biosynthesis of amino acids (ko01230)”, and “starch and sucrose metabolism (ko00500)” were the top three represented pathways among the DEGs between P1 and P4 (Fig. 6C). The “starch and sucrose metabolism (ko00500)”, “amino sugar and nucleotide sugar metabolism (ko00520)”, and “carbon metabolism (ko01200)” were the top three represented pathways among the DEGs between P1 and P5 (Fig. 6D). The “biosynthesis of amino acids (ko01230)”, “carbon metabolism(ko01200)”, and “starch and sucrose metabolism (ko00500)” were the top three represented path ways among the DEGs between P1 and P6 (Fig. 6E).

**Table 3:** KEGG enrichment analysis of common differential genes

Groups	KEGG ID	Term	p-value	Gene number
P1 vs. P2	ko01230	Biosynthesis of amino acids	1.50406E-05	129
	ko01200	Carbon metabolism	1.24518E-05	111
	ko00500	Starch and sucrose metabolism	0.000276537	92
	ko00010	Glycolysis/Gluconeogenesis	0.010246339	87
	ko04075	Plant hormone signal transduction	0.014183796	87
	ko00270	Cysteine and methionine metabolism	0.019448743	63
	ko00940	Phenylpropanoid biosynthesis	0.024968804	62
	ko00520	Amino sugar and nucleotide sugar metabolism	0.030967826	62
	ko04626	Plant-pathogen interaction	0.035170203	55
	ko00710	Carbon fixation in photosynthetic organisms	0.03699648	53
	ko00051	Fructose and mannose metabolism	0.039762764	33
P1 vs. P3	ko00500	Starch and sucrose metabolism	1.40386E-08	77
	ko01200	Carbon metabolism	2.48638E-06	51
	ko04075	Plant hormone signal transduction	1.41496E-06	53
	ko01230	Biosynthesis of amino acids	0.000642265	47
	ko00520	Amino sugar and nucleotide sugar metabolism	0.001897244	45
	ko04626	Plant-pathogen interaction	0.002746559	40
	ko00940	Phenylpropanoid biosynthesis	0.014229602	39
	ko04712	Circadian rhythm - plant	0.018236437	30
	ko03015	mRNA surveillance pathway	0.02455863	29
	ko00350	Tyrosine metabolism	0.031597728	29
	ko00010	Glycolysis / Gluconeogenesis	0.045759682	28
P1 vs. P4	ko01200	Carbon metabolism	1.96805E-08	176
	ko01230	Biosynthesis of amino acids	2.44866E-07	158
	ko00500	Starch and sucrose metabolism	0.000104485	140
	ko04075	Plant hormone signal transduction	0.000446975	121
	ko00010	Glycolysis/Gluconeogenesis	0.001465848	104
	ko00940	Phenylpropanoid biosynthesis	0.003977588	90
	ko03040	Spliceosome	0.007556669	90
	ko00520	Amino sugar and nucleotide sugar metabolism	0.007894432	89
	ko04626	Plant-pathogen interaction	0.015022443	88
	ko04141	Protein processing in endoplasmic reticulum	0.034329967	72
P1 vs. P5	ko00500	Starch and sucrose metabolism	1.5986E-08	94
	ko00520	Amino sugar and nucleotide sugar metabolism	0.000152078	63
	ko01200	Carbon metabolism	0.000964485	63
	ko04075	Plant hormone signal transduction	0.001748152	60
	ko01230	Biosynthesis of amino acids	0.005697883	52
	ko03040	Spliceosome	0.007841245	46
	ko00940	Phenylpropanoid biosynthesis	0.019302214	41
	ko04712	Circadian rhythm - plant	0.024663689	38
	ko00010	Glycolysis / Gluconeogenesis	0.03007295	32
	ko00270	Cysteine and methionine metabolism	0.032804472	32
	ko00900	Terpenoid backbone biosynthesis	0.038755426	31
P1 vs. P6	ko01230	Biosynthesis of amino acids	1.684E-07	227
	ko01200	Carbon metabolism	2.243E-06	223
	ko00500	Starch and sucrose metabolism	0.000124426	213
	ko04075	Plant hormone signal transduction	0.000398301	164
	ko00010	Glycolysis / Gluconeogenesis	0.00842226	142
	ko00520	Amino sugar and nucleotide sugar metabolism	0.010135259	127
	ko00940	Phenylpropanoid biosynthesis	0.010903658	122
	ko03040	Spliceosome	0.018455608	110
	ko04626	Plant-pathogen interaction	0.026782892	101
	ko00270	Cysteine and methionine metabolism	0.028998764	101
	ko00230	Purine metabolism	0.030124232	95
	ko04141	Protein processing in endoplasmic reticulum	0.032667845	79
	ko00710	Carbon fixation in photosynthetic organisms	0.035449624	78
	ko00250	Alanine, aspartate and glutamate metabolism	0.038969985	76

Combining the above results, we suggest that the genes involved in the regulation of cell wall and cell division, plant hormone signaling, starch, and sucrose metabolism pathways, and transcription factors might play vital roles in the tuberous root expansion of PRT. Furthermore, these DEGs were annotated using NR, GO, COG, and KEGG annotations, and we found many DEGs involved in these four pathways, indicating that these three pathways played an important role in the process of Pueraria tuberous root expansion. Therefore, we analyzed the genes related to these pathways.

### Cell wall and cell cycle

The transcriptome data obtained in this study revealed that 54, 22, 57, 45, and 84 genes were related to the cell wall and cell cycle from the DEGs shared by P1 vs. P2, P1 vs. P3, P1 vs. P4, P1 vs. P5, and P1 vs. P6, respectively (Table S4). Among them, LOB domain-containing protein 4—HL_transcript_188442 (*LBD4*), and WUSCHEL-related homeobox 4—HL_transcript_36056 (*WOX4*) were significantly up-regulated at the P3 stage. Moreover, the genes involved in cell division, such as cell division protease (*FtsH*) in the P2 stage, cell division cycle 2 (*CDC2*) family in the P4, P5, and P6 stages, and cell division cycle 48 (CDC*48*) in the cyclin-dependent kinase (*CDK*) in the P4, P5, and P6 stages, were significantly upregulated in the tuberous root expansion stages. The genes involved in cell extension and expansion, including xyloglucan endotransglucosylase/hydrolase (*XTH*), expansin (*EXP*), and extension (*EXT*), were also significantly changed in the tuberous root expansion stage. These results indicate that the formation and development of tuberous roots are dependent on active meristems and cell division.

**Fig. 7:**
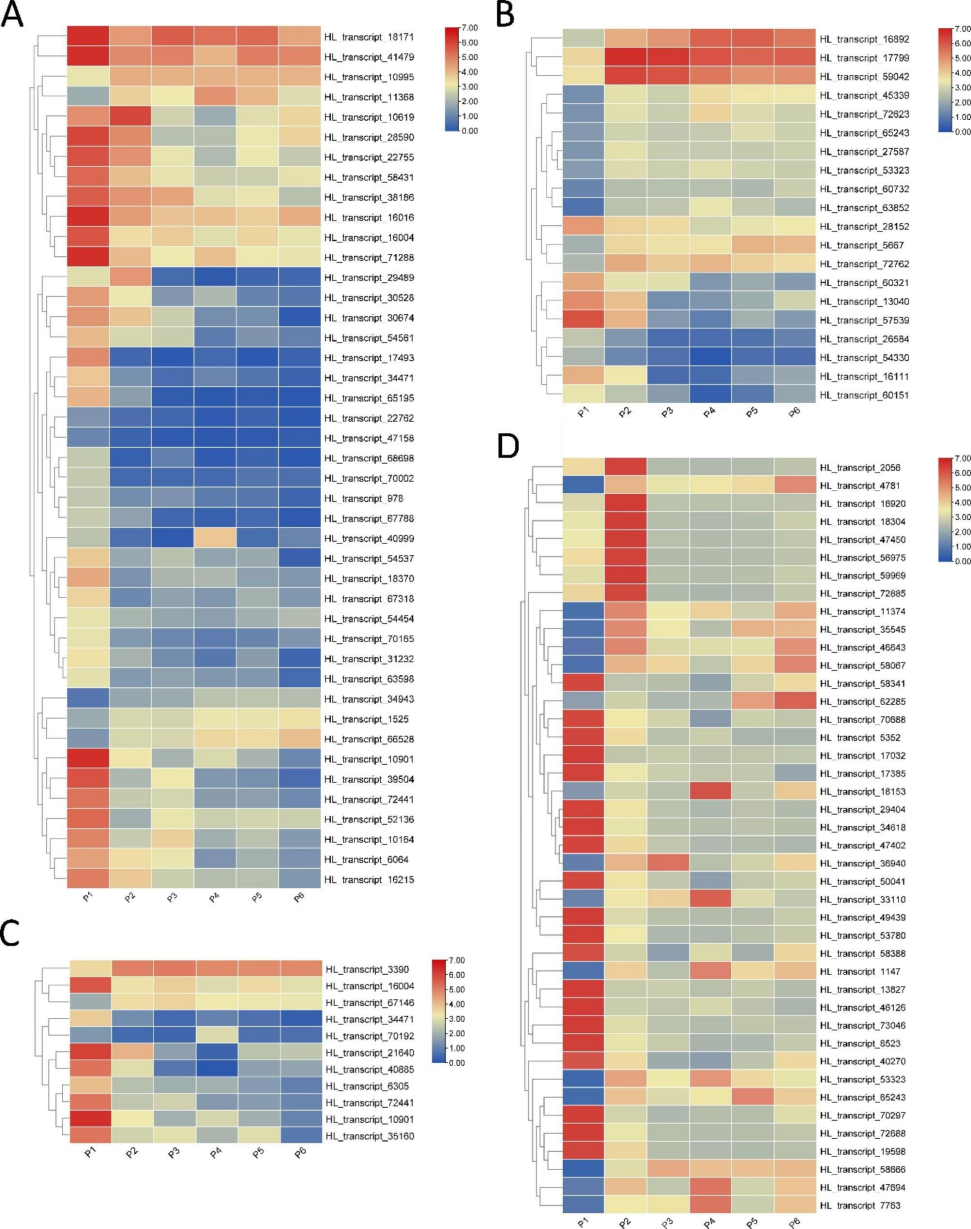
Heatmap of highly expressed DEGs. Every row shows a related gene of tuberous root expansion. Red, yellow, and blue indicate high, middle, and slow levels of mRNA expression, respectively. (**A**) Expression of cell wall and cell cycle; (**B**) Expression of hormone signal; (**C**) Expression of starch and sucrose metabolism; (**D**) Expression of TFs

In total, 43 genes were identified as DEGs shared between the six stages. Among them, 5 genes were upregulated and 38 genes were downregulated during tuberous root development (Fig. 7A). *CDC48*, *XTH*, *EXT*, *EXP*, and *GDSL/SGNH* were the major components of this pathway. HL_transcript_10995 (*CDC48*) was significantly upregulated, and its expression level increased successively in P2–P6 compared to that of the P1 stage of tuberous root expansion. HL_transcript_18370 and HL_transcript_30528 (*XTH*), HL_transcript_72441 (*EXP*), HL_transcript_16215 and HL_transcript_54537 (*EXT)*, and HL_transcript_30674, HL_transcript_54454, and HL_transcript_54561 (*GDSL/SGNH*) were significantly downregulated, and the expression levels decreased successively during this process.

Based on the importance of the P3 stage in the expansion process, we mined 10 genes that were related to the cell wall and cell cycle from the DEGs shared by P3 vs. P1, P3 vs. P2, P3 vs. P4, P3 vs. P5, and P3 vs. P6 (Table 4). In the table, Log_2_FC is the Fold change value, which indicates the ploidy relationship of gene expression. Finally, we screened a total of 2 DEGs related *EXT*2-like (HL_transcript_39504) and pectin acetyl esterase 12-like (HL_transcript_40999), after raising the Log_2_FC value screening threshold from an absolute value greater than 0.5 to 1.

**Table 4:** DEGs enrichment analysis between P3 and other expansion stages

Pathway	Gene ID	Log_2_ Fold Change value				
		P3 vs. P1	P3 vs. P2	P3 vs. P4	P3 vs. P5	P3 vs. P6
Cell wall and cell cycle	HL_transcript_28590	2.543	2.285	0.522	1.009	1.477
	HL_transcript_29489	2.573	5.447	-1.794	-0.576	-0.515
	HL_transcript_30528	2.213	1.701	1.449	-0.968	-1.525
	HL_transcript_30674	0.708	0.885	-1.481	-1.867	-6.566
	HL_transcript_34471	3.610	1.340	1.909	0.627	-1.175
	HL_transcript_39504	1.109	-1.863	-1.922	-2.591	-4.452
	HL_transcript_40999	4.389	2.322	8.046	2.631	3.861
	HL_transcript_68698	0.859	-3.000	-3.180	-1.956	-6.978
	HL_transcript_70002	2.580	-0.511	0.753	0.929	0.592
	HL_transcript_72441	1.239	-0.810	-1.198	-1.488	-2.354
Plant hormone signal	HL_transcript_13040	2.732	2.874	0.696	0.908	1.912
Starch and sucrose metabolism	HL_transcript_21640	3.479	2.498	-2.409	1.118	1.052
	HL_transcript_34471	3.610	1.340	1.909	0.627	-1.175
	HL_transcript_40885	4.234	2.770	-3.222	1.716	1.672
	HL_transcript_72441	1.239	-0.810	-1.198	-1.488	-2.354
Transcription factor	HL_transcript_17032	2.606	-1.344	-1.671	-0.801	-1.244
	HL_transcript_34618	4.533	3.017	-3.484	0.611	-1.629
	HL_transcript_36940	-4.485	-0.949	-0.758	-0.994	-0.629
	HL_transcript_40270	1.624	1.444	-0.597	1.096	1.920
	HL_transcript_47402	2.219	1.508	-1.765	-1.554	-3.635
	HL_transcript_47450	1.416	4.520	-2.271	-0.616	0.862
	HL_transcript_49439	3.331	1.688	-1.580	-1.104	1.255
	HL_transcript_56975	4.763	7.470	0.724	1.473	3.637
	HL_transcript_58388	1.942	1.273	2.402	0.880	2.354
	HL_transcript_70297	3.482	1.877	1.372	0.859	2.711

### Plant hormone signal

We screened for key DEGs in auxin, cytokinin, gibberellin, and abscisic acid signaling during the unexpanded stage (P1) and expanded stages (P2–P6) of Pueraria tuberous roots (Table S5), resulting in 495 DEGs with a significantly different expression, including 274 upregulated genes and 221 downregulated genes. The auxin signal transduction pathway was the most active, followed by the abscisic acid signal transduction pathway. Most genes related to auxin signaling (*AUX*/*IAA* and *ARF*) and cytokinin signaling (*CRE1*) were significantly upregulated. In contrast, genes associated with auxin signaling (*GH3*), cytokinin signaling (*ARR*-*A* and *ARR*-*B*), and abscisic acid signaling (*PP2C*) were significantly downregulated. In this study, the plant hormone signal transduction pathway was one of the most enriched KEGG pathways in the P1 vs. P3 group, indicating that hormone signaling plays a vital role in the P3 stage during tuberous root expansion. In this group, 23 auxin-related genes (*AUX*/*IAA*, *ARF*), 5 cytokinin-related genes (*CRE1*), 7 gibberellin-related genes (*DELLA*, *PYR*/*PYL*), and 8 abscisic acid-related genes (*PP2C*) were upregulated in the tuberous root expansion stage, implying that they may be related to cell expansion during the secondary growth of cambium.

Furthermore, 20 genes were identified as DEGs shared between the six stages. Among them, 12 genes were upregulated and 8 genes were downregulated during tuberous root development (Fig. 7B). The genes *ARF*, *PYL8*, *ARR*, and *SPY* were the major components of this pathway. HL_transcript_72762 (*PYL8*), HL_transcript_60732 (*SPY*), HL_transcript_5667, HL_transcript_45339 (*ARF*), HL_transcript_63852 (*ARF*), and HL_transcript_72623 (*ARF*) were significantly upregulated, and their expression levels increased successively in P2–P6 compared to those in the P1 stage of tuberous root expansion. In contrast, HL_transcript_60151 (*ARR*) was significantly downregulated and its expression levels decreased successively during this process. We only screened a DEG–related ABA catabolic key enzyme (HL_transcript_13040) from the DEGs shared in P3 vs. P1, P3 vs. P2, P3 vs. P4, P3 vs. P5, and P3 vs. P6 (Table 4), but the absolute value of Log_2_FC value does not exceed 1.

### Starch and sucrose metabolism

In this study, changes in the expression of sucrose synthase (*SuSy*) and invertase (*INV*) were active in the tuberous roots of Pueraria in P1 vs. P4 and P1 vs. P6. In P1 vs. P4, 25 *SuSy* genes were significantly upregulated during tuberous root expansion, whereas 8 *INV* genes were significantly downregulated. In P1 vs. P6, 6 *SuSy* genes were significantly upregulated during tuberous root expansion, whereas 18 *INV* genes were significantly downregulated. Furthermore,​ all sucrose phosphate synthase (*SPS*) genes were upregulated during tuberous root expansion before the P3 stage.

In addition, Pueraria tuberous root expansion before the P3 stage was dominated by the upregulation of granule-bound starch synthase (*GBSS*), starch branching enzyme (*SBE*), soluble starch synthase (*SSS*)-related, and isoamylase (*ISA*) genes, and later by the upregulation of *GBSS*, *SBE*, and *ISA* genes. Among them, 9 starch-related genes (1 *GBSS*, 2 *SSS*, 4 *SBE*, and 2 *ISA*) were significantly upregulated in P1 vs. P2, 23 starch-related genes (9 *GBSS*, 4 *SSS*, 6 *SBE*, and 4 *ISA*) were significantly upregulated in P1 vs. P3, 14 starch-related genes (9 *GBSS*, 3 *SBE*, and 2 *ISA*) were significantly upregulated in P1 vs. P4, 10 starch-related genes (3 *GBSS* and 7 *SBE*) were significantly upregulated in P1 vs. P5, and 11 starch-related genes (4 *GBSS*, 10 *SBE*, and 1 *ISA*) were significantly upregulated in P1 vs. P6 (Table S6). These results indicate that many functional genes are involved in the expansion of PRT tuberous roots.

In addition, 11 genes were identified as DEGs shared by the six stages. Among them, two genes were upregulated and nine genes were downregulated during tuberous root development (Fig. 7C). HL_transcript_6305 (*INV*), the major representative of this pathway, was significantly downregulated, and its expression levels decreased successively in P2–P6 compared to those in the P1 stage of tuberous root expansion. We mined 4 genes related to starch and sucrose metabolism from the DEGs shared by P3 vs. P1, P3 vs. P2, P3 vs. P4, P3 vs. P5, and P3 vs. P6 (Table 4). Finally, we screened a total of 2 DEGs related to *β-glucosidase40* (HL_transcript_21640 and HL_transcript_40885), after raising the Log_2_FC value screening threshold from an absolute value greater than 0.5 to 1.

### Transcription factors

In this study, 42 TF genes were identified as DEGs shared by six developmental stages. Among them, 15 TFs were up-regulated, and 20 TFs were down-regulated during the tuberous root expansion (Table S7). *bHLH*, *AP2*, *ERF*, *MYB*, *WRKY*, and *bZIP* TFs were the major represented TF families, which play key regulatory functions in cell differentiation, division and expansion (Fig. 7D). Interestingly, the changes in transcript levels of seven of the eight *bHLH*s showed an “increasing then decreasing” trend, and the expression levels increased in the P2 stages then successively decreased in the P3 ~ P6 stages in PTR. We mined 10 genes related to TFs from the DEGs shared by P3 vs. P1, P3 vs. P2, P3 vs. P4, P3 vs. P5, and P3 vs. P6 (Table 4). Finally, we screened a total of 2 DEGs related to *ERF12* (HL_transcript_47402) and *MYB86*-like (HL_transcript_49439), after raising the Log_2_FC value screening threshold from an absolute value greater than 0.5 to 1.

### Identification of DEGs involved in tuberous root expansion

**Fig. 8:**
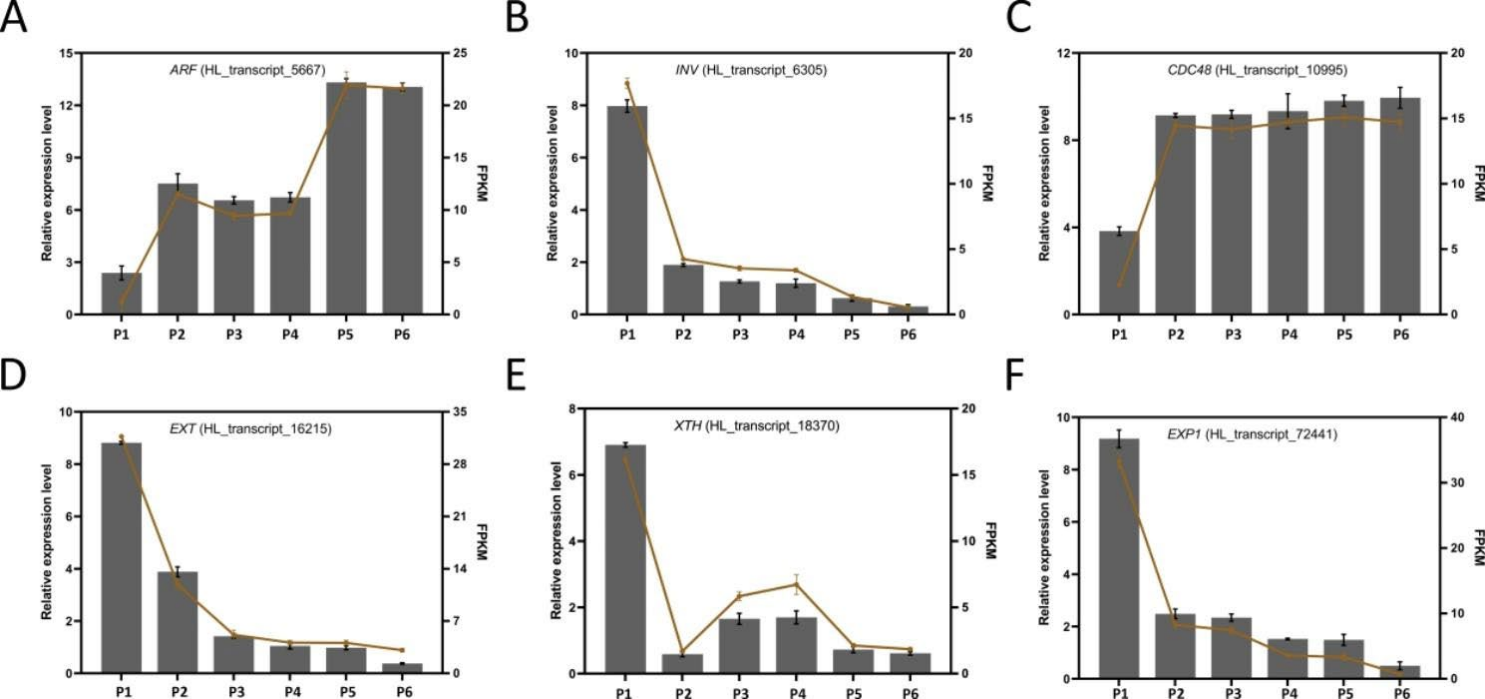
qRT-PCR validation profiles of 6 randomly selected genes. The data were normalized by using *GAPDH* as an internal reference. (**A**) The trend of *ARF* (auxin response factor, HL_transcript_5667) expression in qRT-PCR and transcriptome. (**B**) The trend of *INV* (invertase, HL_transcript_6305) expression in qRT-PCR and transcriptome. (**C**) The trend of *CDC48* (cell division cycle protein 48 homolog, HL_transcript_10995) expression in qRT-PCR and transcriptome. (**D**) The trend of *EXT* (extension, HL_transcript_16215) expression in qRT-PCR and transcriptome. (**E**) The trend of *XTH* (xyloglucan endotransglucosylase/hydrolase, HL_transcript_18370) expression in qRT-PCR and transcriptome. (**F**) The trend of *EXP* (expansion, HL_transcript_72441) expression in qRT-PCR and transcriptome. Black bars represent the data of transcriptome and yellow lines represent the data of qRT-PCR. FPKM values were used to represent the relative expression of genes in the transcriptome. Values are means ± SD (n = 3)

To verify the accuracy of the RNA-seq sequencing results, we selected six DEGs (HL_transcript_5667, HL_transcript_6305, HL_transcript_10995, HL_transcript_16215, HL_transcript_18370, and HL_transcript_72441) involved in cell wall and cell cycle, plant hormone signal, as well as starch and sucrose metabolism for qRT-PCR analysis (Fig. 8). The results showed that the relative expression change trends of qRT-PCR were like that of RNA-seq, demonstrating that transcriptome comparison data were reliable.

## Discussion

The formation and development of tuberous roots directly affect the yield of PTR. Therefore, studying the mechanism of tuberous root expansion is of great importance in improving the yield. The PTR variety used in this study, “Gange No. 1”, is often cultivated as an annual crop, and its entire reproductive period is approximately 260 days. According to the investigation of the traits of Pueraria roots in different reproductive periods, we found that the whole expansion process of “Gange No. 1” could roughly be divided into six stages: the unexpanded, initial-expanded, mid-expanded, mid-late expanded, late-expanded and end-expanded stages. Seedlings were transplanted in a field in the middle of March; vines (stems and leaves) and normal roots grew vigorously from April–June (Fig. 1A). The tuberous roots began to expand in early July (Fig. 1B). Histologically, expansion is caused by the activity of two parts of the meristematic tissue: the primary formative layer (botanically known mainly as the formative vascular layer) and the secondary formative layer (also known as the additional formative layer or paraphyly layer). In this study, we considered that the expansion of the PTR tuberous root was driven by the secondary growth of the xylem (Fig. 2). To play a role in starch accumulation, a large number of thin-walled cells in the secondary xylem divide and differentiate, producing a large number of thin-walled tissues containing starch grains, leading to an increase in tuberous root diameter, which is similar to starch accumulation processes in sweet potato [20] and cassava [23].

Tuberous root expansion relies on an increase in cell volume, in addition to an increase in the number of cells by the division activity of the secondary formative layer [24]. The rate of cell division and the number of cells is usually related to the cell division cycle. For example, genes involved in cell division such as cell division proteases (*FtsH*), cell division control proteins (*CDC48*), and cell cycle proteins (*CYCT1*) were significantly upregulated during radish fleshy root expansion [25]. Additionally, *CDK* genes, which control the cell division cycle, play an essential role in the expansion of tuber mustard [26]. In our study, the P3 stage (165 days) was the crucial dividing point in the expansion process. Before the P3 stage, round vascular cambium forms and the Pueraria tuberous root begins to thicken. After then, we screened a key gene in the transcriptome data, *EXT12*-like, which was expressed at a higher level during the P3 stage compared to other expansion stages, and we suggest that it may be an important regulator of tuberous root thickening in PTR. Tuberous root hypertrophy is dominated mainly by increased cell numbers from the formative layer division activity and expanded cell volume, along with a rapid increase in tuberous root weight (Table 1), which is similar to potatoes [21], sweet potatoes [20], and cassava [27, 28].

Subsequently, the weight gain ratio of the tuberous root increased slowly. Tuberous root expansion is dominated by cell volume longitudinal elongation, accompanied by starch and other substance accumulation, resulting in the continuous enlargement of tuberous roots. We found that many vital genes (Table S4), such as *LBD4* and *WOX4*, were significantly upregulated in the P3 stage. The genes involved in cell division and expansion pathways, such as *FtsH*, *CDC*, *CDK*, *CDC2*, and *CDC48* were significantly altered during tuberous root expansion of PTR, especially in the later stages with significant upregulation. *EXP1* is a cell wall relaxation protein that is an essential regulator of cell wall elongation during plant cell growth [29, 30], and is closely related to cell size. In sweet potatoes, *EXP1* expression levels are higher in the fibrous root stage and lower in the early root storage stage. Antisense *EXP1* plants formed storage roots earlier than wild-type plants. Lignification of the fibrous root mid-column was significantly reduced, providing them a higher potential to develop storage roots, suggesting that *EXP1* plays a suppressive role in the formation of storage roots in sweet potatoes [31]. In this study, we found a similar result that most EXPs had a significant downregulation trend during tuberous root expansion, indicating that downregulation of the expression of these genes could promote tuberous root expansion of PTR.

Changes in the phytohormone content are also involved in the expansion of tuberous roots as a starchy storage organ [13, 32]. These changes are required to alter cell division patterns and stimulate cell enlargement and metabolism to facilitate the storage of starch and proteins. In this study, the IAA, ZR, and GA3 contents of PTR tuberous roots decreased to different degrees during the expansion process, with IAA and GA3 contents decreasing more and ZR decreasing less. All hormone levels decreased to the lowest value at the end-expansion stage (the P6 stage).

Among them, IAA decreased significantly after the beginning of the expansion and remained at a low level at the mid-expansion and maturity stages, indicating that the low concentration of IAA favored tuberous root expansion, which is consistent with the results of IAA content changes in tuber formation in jicama [33]. Meanwhile, the ZR content of PTR was higher during the initiation stage. However, it significantly decreased with tuberous root expansion, indicating that high concentrations of cytokinin facilitate the initiation of primordium differentiation, which is consistent with observations in onion bulbs [34]. In addition, GA3 content continuously decreased during the entire process of PTR tuberous root expansion. A previous study has shown that a decrease in GA content in potato tubers induces tuber formation [35] while planting under high GA concentrations prevents tuber formation [36]. Similar results were also obtained during the development of sweet potato storage roots [37]. These reports supported our speculation that GA might also be an inhibitor of PTR tuberous root expansion. In transcriptome data, the plant hormone signal transduction pathway was one of the most enriched KEGG pathways in the P1 group compared to the P3 group. Auxin plays a vital role in cambium cell proliferation and cell expansion [38], maintaining the meristem state of cambium cells, and increasing the number of xylem elements [39]. Studies conducted on sweet potato, radish, *Rehmannia glutinosa* (Di Huang), and *Callerya speciosa* (Niu Dali) reported that the expression of auxin-related genes is significantly upregulated during tuberous root expansion stages [40–42]. In our study, 23 auxin-related genes (10 *AUX*/*IAA* and13 *ARF*) were upregulated in the P1 vs. P3 group (Table S6), indicating that they might be related to cell expansion during the secondary growth of the tuberous root. Cytokinin is involved in the proliferation and development of cambium cells. The expression of auxin-related genes reached the highest level in the rapid growth stage of the tuberous root, which was related to the development and formation of the tuberous root/tuber [39, 43–45]. In this study, five cytokinin-related genes (HL_transcript_22447, HL_transcript_59990, HL_transcript_25588, HL_transcript_1520, and HL_transcript_43612) were significantly upregulated during tuberous root expansion, suggesting that cytokinin might promote tuberous root expansion by enhancing cambium development.

In addition, ABA, the classical antagonistic hormone of GA, showed the lowest value at the P3 stage during PRT expansion. From the transcriptome data, we found that most of the *PP2C* and *ABF* genes were significantly downregulated in the ABA-responsive pathway during tuber formation. In contrast, *PYR*/*PYL* genes were significantly upregulated, suggesting that ABA accumulation during PRT expansion may have a negative feedback regulation on *PYR*/*PYL*. Additionally, *PP2C* expression was enhanced by *ABF* genes, which is consistent with the literature on ginger rootstock expansion [46]. Although it is known that these hormone-related genes play vital roles during the tuberous root expansion stage, further in-depth studies of their systematic network are needed to assess their role in tuberous root expansion.

The fleshy rootstock becomes an organ for storing carbohydrates (sugars, starch, etc.), accompanied by material and energy changes during development. As a result, the ability to synthesize, accumulate, and metabolize carbohydrates and proteins is an essential criterion for determining the expansion ability of metamorphic rootstocks [47]. Starch is the material basis for the expansion of PTR tuberous roots and it usually accounts for 15–46% of fresh matter weight [48]. In our study, starch grains accumulated significantly with the growth of PTR tuberous roots (Fig. 2), and sugar, sucrose, and starch contents gradually increased, along with total flavonoid and puerarin accumulation (Fig. 3). Combined with the transcriptome data, we found that the pathways “carbon metabolism” and “sucrose and starch metabolism” were present throughout the tuberous root expansion of PRT. These results indicate that carbohydrate metabolism play a vital role in the tuberous root expansion of PTR, which is consistent with our phenotypic data. During the formation and expansion of PTR storage organs, the expression of most *SPS*, *SS*, *GBSS*, *SSS*, *SBE*, and *ISA* genes was significantly upregulated during the process of PRT tuberous root expansion. Most *INV* genes were significantly downregulated during this process. In contrast, the a*-amylase* and *b-amylase* genes first decreased and then increased (Table S6), which is similar to patterns found in most tuber species, such as potato [49], sweet potato [50], radish [51], and cassava [52]. Sucrose, the main product of photosynthesis, supports plant growth and development. Sucrose synthesis in plants is mainly regulated by three enzyme genes: *SPS*, *SuSy*, and *INV*. *SPS*, the primary source of sucrose synthesis activity [53], was upregulated during PRT tuberous root expansion. As a result, we suggest that *SPS* might play an essential role in PRT tuberous root expansion, consistent with previous studies in radishes wherein upregulation of *SPS* played a significant role in the thickening stage of radish taproot [54]. In addition, high transcript levels of *β-glucosidase40* in the P3 stage could be related to the increased fiber during expansion, and their regulatory mechanism should be studied in the next step.

The sucrose synthase acts as a bidirectional regulator that synthesizes sucrose and breaks it down into glucose and fructose [55]. Combined with the previous sugar content assay data, *SuSy* genes upregulated in the pre- and mid-expansion stages of PTR might exercise mainly a catabolic function, consistent with the increase in glucose observed during the P2–P3 stages. In contrast, sucrose synthase primarily exerts a synthetic function during the later phase, consistent with the rapid increase in sucrose during the P4–P6 stages. In addition, INV positively regulates fructose and glucose contents in turnip fleshy roots, thereby accelerating the accumulation of biomass in the roots [56, 57]. Our results most closely resembled those of previous studies on sweet potatoes, which found that INV rapidly decreases to an undetectable level during storage root development [58]. Based on these results, we speculate that INV might not be the primary regulator of glucose and fructose content in PTR tuberous root expansion, and might be related to other genetic characteristics of PTR. Further studies are needed for clarification.

TFs play an important role in the regulation of plant growth and development and secondary metabolism. In this study, we identified 15, 27 TFs that were significantly up-and down-regulated in the tuberous root expansion process. bHLH, AP2, ERF, MYB, WRKY, and bZIP were the main types of TFs, which is different from the results in sweet potato [20] and potato [21], probably because of species differences. Among these TFs, ERF12 and MYB86 are two transcription factors that are prominent in the P3 stage, indicating they may be involved in the regulation of the tuberous root expansion in PTR, but further functional identification studies were needed to confirm the functions of these potential genes.

**Fig. 9:**
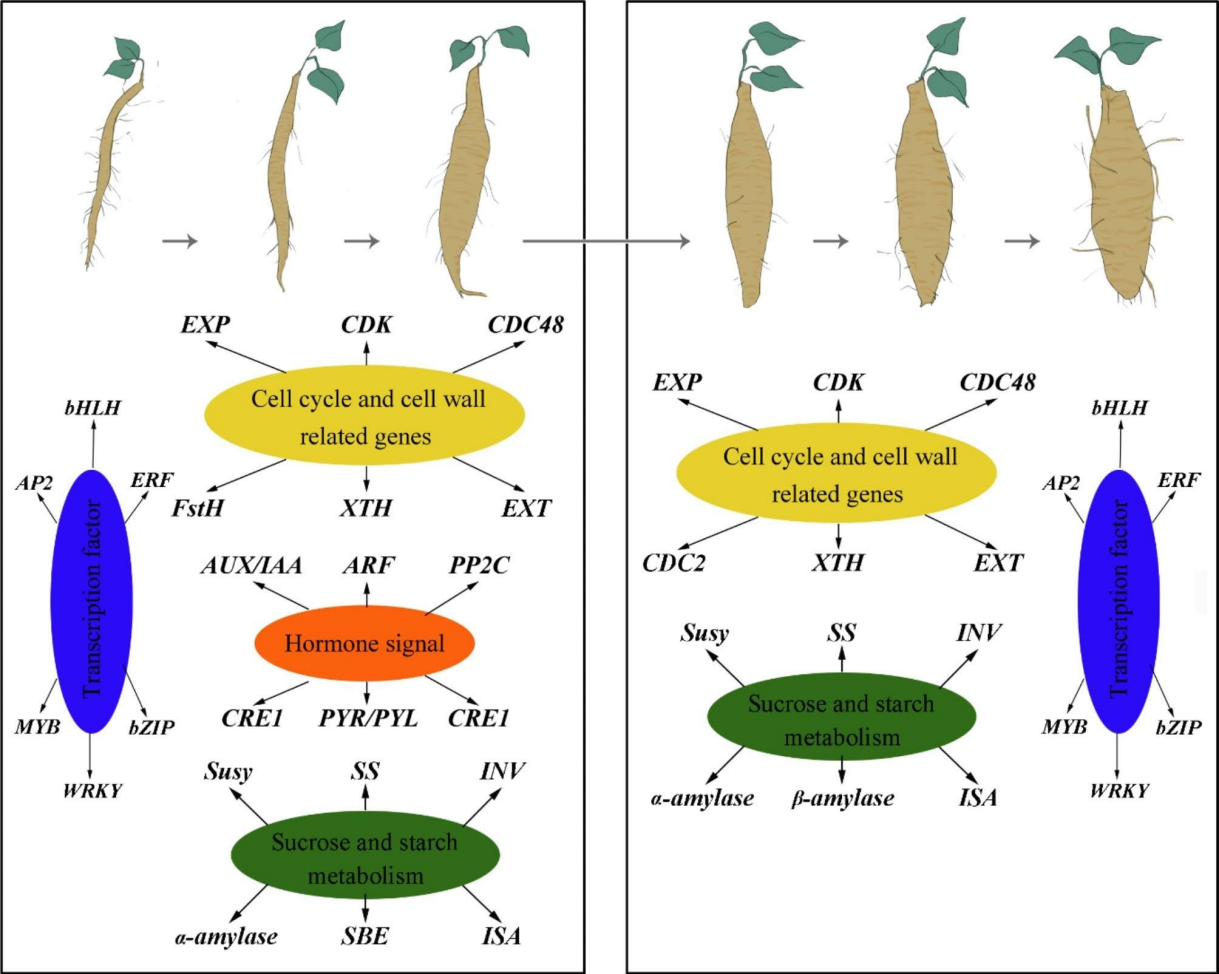
A hypothetical model of regulatory network related to tuberous root expansion in PTR

PTR tuberous root expansion is a complex regulatory process affected by many factors. In this study, through transcriptome analysis combined with phenotype and physiological test results, a hypothetical model of the PTR tuberous root expansion regulatory network was proposed (Fig. 9). The cells in the vascular cambium divide and expand continuously to produce secondary xylem and phloem, resulting in the expansion of tuberous roots. Cell proliferation and size are regulated by several signal transduction pathways (hormone signaling) and metabolic processes (cell wall, sucrose, and starch metabolism). Several genes, including auxin-related genes (*Aux*/*IAA*, *ARF*, *CRE1*, and *PYR*/*PYL*), were highly expressed to promote cell differentiation, division, expansion, sucrose, and starch accumulation in the secondary structure. Additionally, *FtsH*, *CDC*, *CDK*, *XTH*, *EXP*, and *EXT* were involved in the extension and expansion of cell division. Finally, *SuSy*, *SPS*, *SSS*, *GBSS*, and *SBE* were involved in the hydrolysis of sucrose and starch synthesis.

## Conclusions

Integrated phenotypic, physiological, and transcriptomic analyses performed here revealed that the P3 stage was a vital boundary point in the expansion period of tuberous roots of PTR. There were 17,441 differential genes shared between the unexpanded (P1) and expanded (P2–P6) stages of PRT. KEGG pathway enrichment analysis revealed that cell wall and cell cycle, plant hormone signal transduction, and starch and sucrose metabolism were the top pathways participating in this process. The qRT-PCR analysis suggested that *ARF*, *INV*, *CDC48*, *EXT*, *XTH*, and *EXP* might play a key role in tuberous root expansion in PTR. Based on the above results, we propose a hypothetical model of a genetic regulatory network associated with tuberous roots in PTR (Fig. 9). The tuberous root expansion of PTR was mainly attributed to cell differentiation, division, expansion, and starch accumulation, all of which are regulated and promoted by specific signal transduction pathways and metabolic processes. These findings not only provide novel insights into the molecular regulation mechanism of tuberous root expansion but also support the theoretical basis for genetic improvement of PTR, which may help enhance PTR yield for food and medicinal purposes.

## Methods

### Plant materials

The local variety “Gange No. 1” belongs to an annual PTR, and the entire reproductive period is approximately 260 days. The entire planting process of “Gange No. 1” was performed at the Jiangxi Agricultural University Experimental Station in Nanchang (28º 09’ N, 116º 35’ E), Jiangxi Province, China. Pueraria seedlings were transplanted on March 18, 2021. Tuberous roots were collected at 10 am each time across 6 developmental stages at 105 (June 29), 135 (July 29), 165 (August 29), 195 (September 29), 225 (October 29), and 255 days (November 29) post-transplant, named P1, P2, P3, P4, P5, and P6 stages, respectively. For each sample time, three independent biological replicates of tuberous roots were collected. Root samples were frozen immediately in liquid nitrogen and stored at − 80℃ until further processing.

### Phenotype assessment

We adopted a bamboo shelf with a nylon mesh cultivation mode. We used single-stem pruning for each PTR plant during cultivation and retained one tuberous root after outcropping. In addition, we used high monopoly cultivation, with the following measurements: width of the ridge bottom (1.1 m), the width of the ridge (60 cm), the height of the ridge (50 cm), and the spacing of the plants (30 cm apart) with planting 1400 seedlings per acre.

For each stage, root diameter, length, and weight were measured and recorded using a ruler and an electronic scale. We measured the root weight after cleaning and drying and recorded the root diameter and length data by measuring the transverse diameters and the most significant longitudinal lengths of the roots. Finally, we performed statistical analyses using SPSS software.

### Histological analysis of tuberous roots

Since the P3 stage is an important boundary point during PTR expansion, we collected tuberous roots at three typical expansion stages, P1, P3, and P6, as materials for cytological characterization. Next, the sections were fixed, embedded, cut into slices, and stained according to conventional paraffin sectioning methods. Slices were observed and photographed using a microscope (Nikon ECLIPSE 80i, Tokyo, Japan). Voucher specimens of three PTR developmental stages (P1, P3, and P6) were deposited in the Herbarium of Agronomy College, Jiangxi Agricultural University.

### Determination of carbohydrate content

The samples were ground and placed in 50-mL centrifuge tubes to test the main carbohydrate content. The glucose content was determined using the glucose oxidase method [59]. The resorcinol method was used to determine the fructose and sucrose contents [60]. The total starch content was determined using the anthrone sulfate method [61]. The total soluble sugars were determined using the anthrone sulfate method [62]. The reducing sugars were determined colorimetrically using 3, 5-dinitro salicylic acid (DNS) [63].

### Determination of hormone contents

We modified the methods used for the extraction and purification of endogenous hormones (IAA, ZR, GA3, and ABA) from those described by Yang et al. [64] and Wang et al. [65]. Culture tissue samples (approximately 0.5 g) were ground using an ice-cooled mortar in 5 mL 80% (v/v) methanol extraction medium containing 1 mM butylated hydroxytoluene and 100 mg polyvinylpyrrolidone (PVP) per gram of fresh material. The extract was incubated at 4 °C for 4 h and centrifuged at 1000 × *g* for 15 min at the same temperature. The supernatant was passed through a Chromosep C18 column (C18 Sep-Park Cartridge, Waters Corp., Milford, MA, USA) that was prewashed with 1 mL of 80% methanol. Hormone fractions were eluted with 5 mL of 100% methanol, 5 mL of ether, and 5 mL of 100% methanol, then dried under N_2_, and dissolved in 2 mL of PBS (pH 7.5) containing 0.1% Tween 20 and 0.1% gelatin before using ELISA kits from China Agricultural University (Beijing, China) to estimate hormone levels.

### RNA extraction and transcriptome sequencing

Total RNA was extracted according to the instructions in the TRIzol reagent kit (Thermo Fisher Scientific, Waltham, MA, USA). The RNA concentration was measured using a NanoDrop 2000 spectrophotometer (Thermo Fisher Scientific). RNA integrity was assessed using an RNA Nano 6000 assay kit from the Agilent Bioanalyzer 2100 system (Agilent Technologies, Santa Clara, CA, USA). The RNA samples were stored at − 80ºC until use. First, mRNA was purified from the total RNA using polyToligo-attached magnetic beads and then broken into short fragments. Random hexamers and RNA fragments were used to prime cDNA synthesis. After purification and connection with adapters, a cDNA library was constructed using PCR amplification. The length of the insert sequence was verified using an Agilent Bioanalyzer 2100 system and the library was quantified using an ABI StepOnePlus Real-Time PCR System. Finally, the qualified cDNA library was sequenced using the Illumina HiSeq 2000 system.

### Transcriptome analysis

Low-quality reads (more than 20% of bases with quality ≤ 10) and reads with adapters (more than 5% unknown nucleotides) were filtered to generate only clean reads for data collection. *De Novo* assembly and redundant sequence removal were performed using Trinity and Tgirl, respectively.

The resultant transcripts were searched against the NCBI nonredundant nucleotide (Nt), the NCBI nonredundant protein sequences (Nr), and SwissProt protein databases for functional annotation using the BLAST algorithm with an E-value cutoff of 1e-5. The available categories of these unique sequences were further analyzed using Clusters of Orthologous Groups of Proteins (GO), Gene Ontology (GO), and Kyoto Encyclopedia of Transcripts and Genome (KEGG) databases using the BLAST and Blast2 GO programs. The clean reads were mapped to the reference using Bowtie 2 to estimate the transcript expression profiles. The expression levels were calculated as fragments per kilobase of exon per million fragments (FPKM) using RSEM. Candidate transcripts involved in swollen root formation were selected from previous reports and databases with FPKM values of the transcripts converted to log10 values (FPKM ≥ 5). They were visualized in a heatmap to identify different expression profiles between P1 vs. P2, P1 vs. P3, P1 vs. P4, P1 vs. P5, and P1 vs. P6.

### Identification of candidate genes involved in tuberous root expansion

The expression of the six Unigenes identified by RNA-seq was validated using RT-qPCR. Gene-specific primers were designed based on Unigene sequences (Table 5). The 18SrRNA genes were amplified as an endogenous loading control. Finally, the expression of each gene was confirmed using at least three rounds of independent qRT-PCR.

**Table 5:** The primers of 6 DEGs validated by RT-qPCR analysis

Gene ID	Primer sequence (5’-3’)	Up-Down-Regulation	Gene name
HL_transcript_5667	GGATTCTCTGTGCCTCGTCGC AAGTGTCTTCGTGGCTGCCC	up	*ARF*
HL_transcript_6305	ACGGCGTTAAGGTGGACGTG TAGAGCGCGTCCGTGTTGTG	down	*INV*
HL_transcript_10995	AGCAGGCGGTTCGTGAAGAC TCACCACCTCCCGTGTCAAGA	up	*CDC48*
HL_transcript_16215	GGAGTCCGATGTGGATGGCG CCGACAACTCGCTCGGAAACT	down	*EXT*
HL_transcript_118370	AAGGTGCGTGTGATGATGGT TACCCATACCGACAGCTCCA	down	*XTH*
HL_transcript_72441	TCAACGCGATCACCGCACAA GCAGCGTTGATCACACTGTCCA	down	*EXP*

### Statistical analysis

The experimental data were analyzed in triplicate. The data were analyzed using SPSS 17.0. The measurement data were expressed as mean ± standard deviation (SD). Duncan’s multiple range test was used for inter-group and intra-group comparison. *p* < 0.05 denotes statistical significance.

## Electronic supplementary material

Below is the link to the electronic supplementary material.

Supplementary Material 1

Supplementary Material 2

Supplementary Material 3

Supplementary Material 4

Supplementary Material 5

Supplementary Material 6

Supplementary Material 7

Supplementary Material 8

## Data Availability

RNA sequencing data have been deposited in the sequence read archive - SRA (https://www.ncbi.nlm.nih.gov/sra/PRJNA923481). Data supporting the findings of this study are available at https://github.com/xiaoxf820/my-document.

## Abbreviations

DEGs: Differentially expressed genes
IAA: Auxin
CTK: Cytokinin
ABA: Abscisic acid
GA: Gibberellin
LBD: LOB domain-containing protein
WOX4: WUSCHEL-related homeobox 4
XTH: Xyloglucanendotrans-glucosylase/hydrolase
FtsH: Cell division protease
CDC2: Cell division cycle2-like protein
CDC20: Cell division cycle20-like protein
CDC48: Cell division cycle protein 48 homolog
CDK: Cyclin-dependent kinase
CDKI: Cyclin-dependent kinase inhibitor
EXP: Expansin
EXT: Extension
AUX1: Auxin influx carrie
AUX/IAA: Auxin-responsive protein IAA
GH3: Auxin responsive GH3 gene family
TIR1: Transport inhibitor response
SAUR: Small auxin up RNA
CRE1: Cytokinin receptor
ARR-B: Two-component response regulator ARR-B family
ARR-A: Two-component response regulator ARR-A family
GID2: F-box protein GID2
PIF3: Phytochrome-interacting factor 3
PYR/PYL: Abscisic acid receptor
PP2C: Protein phosphatase 2C
ABF: ABA responsive element binding factor
SuSy: Sucrose synthase
SPS: Sucrose phosphate synthase
INV: Invertase
SS: Starch synthase
GBSS: Granule-bound starch synthase
SSS: Soluble starch synthase
SBE: Starch branching enzyme
AGPase: ADP-glucose pyrophosphorylase
ISA: Isoamylase
