# Supplementary material for: Transcriptome profiling reveals the genes involved in tuberous root expansion in Pueraria (Pueraria montana var. thomsonii)
Source: BMC Plant Biol. 2023 Jun 26;23:338. doi: 10.1186/s12870-023-04303-x (PMC10291767; doi:10.1186/s12870-023-04303-x)
Supplement: Supplementary file 1 — Supplementary Material 1 [file 12870_2023_4303_MOESM1_ESM.docx]

### Supplementary Tables

**Table S1.** All DEGs in the six comparison groups

**Table S2.** 386 DEGs in six tuberous root expansion stages

**Table S3.** All DEGs in the P1 vs. P2, P1 vs. P3, P1 vs. P4, P1 vs. P5, and P1 vs. P6 group

**Table S4.** All DEGs of cell wall and cell cycle pathway between the P1 stage and P2 ~ P6 stages

**Table S5.** All DEGs of plant hormone signal transduction between the P1 stage and P2 ~ P6 stages

**Table S6.** All DEGs of sucrose and starch metabolism pathway between the P1 stage and P2 ~ P6 stages

**Table S7.** All DEGs of transcription factors between the P1 stage and P2 ~ P6 stages
